# Mental health in clinically referred children and young people before and during the Covid-19 pandemic

**DOI:** 10.1007/s00787-022-02115-2

**Published:** 2022-12-17

**Authors:** Kapil Sayal, Christopher Partlett, Anupam Bhardwaj, Bernadka Dubicka, Tamsin Marshall, Julia Gledhill, Colleen Ewart, Marilyn James, Alexandra Lang, Kirsty Sprange, Alan Montgomery

**Affiliations:** 1 School of Medicine, University of Nottingham, Nottingham, UK; 2 Institute of Mental Health, Nottinghamshire Healthcare NHS Foundation Trust, Nottingham, UK; 3 Cambridgeshire and Peterborough NHS Foundation Trust, Cambridge, UK; 4 University of Manchester, Manchester, UK; 5 Berkshire Healthcare NHS Foundation Trust, Reading, UK; 6 Central and North West London NHS Foundation Trust, London, UK; 7 Faculty of Engineering, University of Nottingham, Nottingham, UK

**Keywords:** Covid-19, Children and young people, Mental health, Lockdown, School closures, School re-openings, STADIA

## Abstract

The Covid-19 pandemic and mitigation approaches, including lockdowns and school closures, are thought to have negatively impacted children and young people’s (CYP) mental health. However, the impact for clinically referred CYP is less clear. We investigated differences in the mental health of CYP referred to specialist Child and Adolescent Mental Health Services (CAMHS) before and since the onset of the pandemic. Using baseline data (self- and parent- completed Mood and Feelings Questionnaire and Strengths and Difficulties Questionnaire) from an ongoing RCT (STADIA; ISRCTN: 15748675) in England involving 5–17-year-olds with emotional difficulties recently referred to CAMHS (non-urgent referrals), with repeated cross-sectional comparisons of CYP (*n* = 1028) recruited during 5 different time  periods: (1) Before schools were closed (Group 1 (pre-pandemic); *n* = 308; 27.08.2019–20.03.2020). (2) Early pandemic period until schools fully re-opened, which included the first national lockdown, its easing and the summer holidays (Group 2 (in-pandemic); *n* = 183; 21.03.2020–31.08.2020). (3) The following school-term—schools fully re-opened and remained open, including during the second national lockdown (Group 3 (in-pandemic); *n* = 204; 01.09.2020–18.12.2020). (4) Schools closed as part of the third national lockdown (Group 4 (in-pandemic); *n* = 101; 05.01.2021–07.03.2021). (5) Schools re-opened and remained open, until the school summer holidays (Group 5 (in-pandemic); *n* = 232; 08.03.2021–16.07.2021). Most CYP scored above cutoff for emotional problems and depression, with three-quarters meeting criteria for a probable disorder (‘caseness’). The groups did not differ on parent-rated mental health measures. However, self-rated emotional problems, depression, functional impairment and caseness appeared to be higher amongst participants recruited in the two periods following school re-openings. In particular, functional impairment and caseness were greater in Group 5 compared with Group 2. Although symptom severity or impairment did not change in the initial pandemic period, self-reported difficulties were greater during the periods after schools re-opened. This suggests possible greater stresses in the adjustment to re-starting school following recurrent lockdowns and school closures.

## Introduction

The Covid-19 pandemic and population-level approaches to manage it (such as national lockdowns and school closures) have led to concerns about the potential negative impacts on the mental health of children and young people (CYP) [1]. For example, at the beginning of the pandemic Young Minds carried out a survey in the United Kingdom (UK) with over 2000 young people with a history of mental health needs and the majority said that the pandemic had made their mental health worse [2]. Qualitative studies have also highlighted the impact on wellbeing and mental health, including the onset or worsening of anxiety, depression and suicidal thoughts, relating to social isolation and loneliness [3–5]. Several longitudinal studies have looked at changes in CYP’s mental health over time by comparing measures completed before and since the onset of the pandemic, but only in community-based samples. For example, a UK study of 7–11-year-olds (*n* = 168) suggested increases in self-reported depression but not parent-rated emotional symptoms [6]. By contrast, a UK birth cohort study of 11–12-year-olds (*n* = 202) found increases in both self-reported and parent-rated depression symptoms [7]. Furthermore, an Australian study of 13–16-year-olds (*n* = 248) found increases in self-reported depression and anxiety symptoms [8] and a study from the United States (US) highlighted elevated trajectories relating to self-reported depression and anxiety symptoms (sample mean age 16 years; *n* = 136) [9]. In terms of potentially meeting criteria for a probable mental health disorder (‘caseness’), a national survey of 5–16-year-olds in England suggested that the population prevalence increased from 11% in 2017 to 16% in July 2020 [10]. In keeping with this, from an international perspective, a meta-analysis using symptom severity data (i.e. reflecting elevated levels of symptoms scoring above cutoffs) suggests that the population prevalence of depression and anxiety amongst CYP may have doubled since the onset of the pandemic [11].

However, it is unclear to what extent these findings might generalise to CYP who have been referred to specialist mental health services. To our knowledge, no studies have focussed on CYP with emotional difficulties who have been recently referred to Child and Adolescent Mental Health Services (CAMHS) with a view to systematically collecting information about mental health symptomatology, during the immediate pre-pandemic and in-pandemic periods. Based on the literature from community samples [6–9], it might be expected that those referred since the onset of the pandemic have greater symptom severity. However, there have been a number of distinct phases since the pandemic started, particularly in relation to school closures and re-openings. A direct comparison of those referred either in the pre-pandemic or at any point in the in-pandemic period might miss the possibility of differential impacts related to these distinct phases in the pandemic timeline. Improved knowledge and understanding about the possible differential impacts associated with these phases could help key adults (such as parents, teachers, referrers and mental health professionals) in guiding them how to best support affected CYP.

We report baseline data collected during the first 22 months of a UK-based randomised controlled trial (RCT) which commenced recruitment in August 2019 and gathered information from the parent and/or young person. By using the same measures over a 22-month period spanning both pre-pandemic and in-pandemic periods, we aim to investigate differences in the mental health of CYP who were referred before or during different phases since the onset of the pandemic.

## Methods

### Sample and data collection

STADIA (STandardised DIagnostic Assessment for children and young people with emotional difficulties) is a multi-site RCT investigating the effectiveness and cost-effectiveness of a standardised diagnostic assessment tool (the Development and Wellbeing Assessment; DAWBA) [12], compared with assessment as usual, in CAMHS in England [13]. The intervention arm is completion of the DAWBA in addition to assessment as usual; the comparator arm is assessment as usual. CAMHS is a multi-disciplinary secondary care mental health service for CYP with mental health difficulties—referrals can be made by professionals and some services also accept self-referrals. The trial focus is on 5–17-year-olds with emotional difficulties referred to CAMHS (excluding urgent/emergency referrals—referrals deemed to be urgent/emergency according to local risk assessment procedures require expedited clinical contact and assessment) [13]. Potentially eligible participants were approached following referral receipt and, after providing informed consent, completed the STADIA trial baseline questionnaires within 14 days. For 5–10-year-olds, the parent/carer is the primary and only participant providing data i.e. completing study questionnaires. For 11–15-year-olds, the parent/carer is the primary participant and, with their permission, the young person (secondary participant) can also provide data. By contrast, 16–17-year-olds are primary participants and, with their permission, their parent/carer (secondary participant) can also provide data. For further information about the trial procedures, please see the published protocol [13]**.** Recruitment to the trial commenced in August 2019, initially across 5 large National Health Service (NHS) mental health trusts (sites) across England. In this paper, we focus on baseline data from participants recruited and randomised at these sites between 27.08.2019 and 16.07.2021. Of the eligible referrals that were screened up till July 2021, 23% of participants were successfully randomised into the trial. Ethics Committee approval: South Birmingham Research Ethics Committee (Ref. 19/WM/0133).

### Measures

The following measures were used:

1. The Mood and Feelings Questionnaire (MFQ) is a valid and reliable 33-item measure (score range 0–66) of depression in CYP—each item is answered and scored on a 3-point scale (‘not true’ = 0, ‘somewhat true’ = 1 point, ‘true’ = 2 points) and scores of 27 or above are indicative of depressive disorder [14–16]. The MFQ has very well-established test–retest reliability and convergent and concurrent validity [17]. In our sample, the internal consistency was excellent (Cronbach’s alpha of 0.92 for self-report and 0.93 for parent-report).
2. The Strengths and Difficulties Questionnaire (SDQ) is a widely used 25-item emotional and behavioural measure for CYP, with very well-established reliability and validity [17–19]. It has 5 sub-scales (range 0–10) for emotional problems, conduct problems, hyperactivity/inattention, peer problems and prosocial behaviour, with a total difficulties score based on the first four sub-scales (0–40). In our sample, the internal consistency was good (Cronbach’s alpha of 0.78 for self-report and 0.86 for parent-report). A separate impairment scale (0–10) enquires about distress and impact on home life, friendships, learning and leisure activities [18]. In our sample, the internal consistency was moderate-good (Cronbach’s alpha of 0.66 for self-report and 0.73 for parent-report). Each scale has validated cut-points reflecting approximately 10% of children in community samples (www.sdqinfo.com) and cutoff scores are strongly associated with an increased probability of mental health disorder [19]. To illustrate clinical significance, we also describe the proportion of CYP meeting both symptom (total difficulties) and impairment criteria for probable disorder (i.e. ‘caseness’)—this is associated with an over 20-fold increased likelihood of having a mental health disorder [20]. Unlike the MFQ (which was completed pre-randomisation), the SDQ was completed immediately post-randomisation as it forms the first component of the DAWBA tool for those randomised to the intervention arm.

### Covid-19 key dates in England

**Fig. 1:**
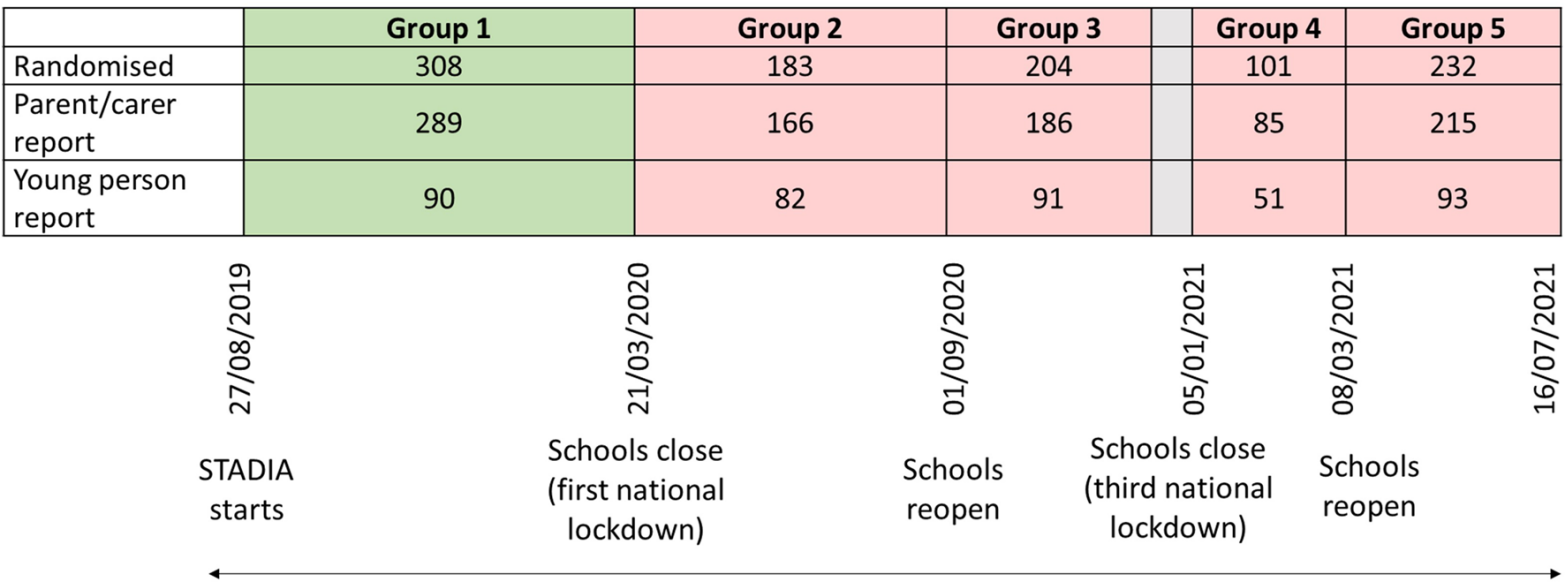
Timeline diagram: Covid-19 key dates in England with sample size of parent- and self-completed questionnaires during the different time periods. Group 1: pre-pandemic: 27.08.2019–20.03.2020. Group 2: early pandemic and schools closed: 21.03.2020–31.08.2020. Group 3: schools re-open: 01.09.2020–18.12.2020. Group 4: schools closed as part of the 3rd national lockdown: 05.01.2021–07.03.2021. Group 5: schools re-open, until summer holidays (mid-July): 08.03.2021–16.07.2021

As shown in the timeline diagram (Fig. 1), schools across England were closed on 20.03.2020 in advance of the first national lockdown. Lockdown restrictions were gradually eased and all schools re-opened in September 2020 following the school summer holidays, remaining open during the brief second national lockdown in November 2020. However, schools were closed again (05.01.2021–07.03.2021) as part of the third national lockdown. To assess whether there was any differential impact associated with specific phases since the start of the pandemic, participants randomised after 20.03.2020 were categorised into four distinct in-pandemic groups based on key dates as outlined below and in Fig. 1.

### Analysis

We compared the scores on self- and parent-completed questionnaires between five groups (total *n* = 1028) of CYP who were referred and randomised in the trial:

1. Before schools were closed—Group 1 (pre-pandemic); *n* = 308 (reflecting 289 parent-completed questionnaires and 90 young person-completed questionnaires); participants randomised between 27.08.2019 and 20.03.2020.
2. During the early pandemic period until schools fully re-opened, which included the first national lockdown, its easing and the summer holidays—Group 2 (in-pandemic); *n* = 183 (reflecting 166 parent-completed questionnaires and 82 young person-completed questionnaires); participants randomised between 21.03.2020 and 31.08.2020.
3. During the following school-term (schools had fully re-opened and remained open, including during the second national lockdown)—Group 3 (in-pandemic); *n* = 204 (reflecting 186 parent-completed questionnaires and 91 young person-completed questionnaires); participants randomised between 01.09.2020 and 18.12.2020.
4. During the period that schools were closed as part of the third national lockdown—Group 4 (in-pandemic); *n* = 101 (reflecting 85 parent-completed questionnaires and 51 young person-completed questionnaires); participants randomised between 05.01.2021 and 07.03.2021.
5. During the subsequent period when schools re-opened and remained open, until the school summer holidays—Group 5 (in-pandemic); *n* = 232 (reflecting 215 parent-completed questionnaires and 93 young person-completed questionnaires); participants randomised between 08.03.2021 and 16.07.2021.

Continuous scores were summarised in terms of the mean and standard deviation in each of the groups. Categorical data were summarised in terms of frequency counts and percentages in each of the groups. No formal statistical comparisons were made because of the large number of comparisons (risk of chance findings), exploratory nature of the analyses and to enable a focus on clinically relevant differences. Furthermore, formal analyses that adjusted for baseline characteristics of the participants were not meaningfully different to unadjusted analyses, providing further support for the descriptive approach to the analyses.

## Results

### Sample characteristics

Demographic baseline characteristics (Table 1) of the CYP were similar across the five groups (pre-pandemic and in-pandemic) in terms of age (mean (s.d.) 11.9 (3.1) years), sex (57% female) and ethnicity (86% white). Around one-third (32%) of the sample had previously been referred to CAMHS.

**Table 1:** Child/young person baseline characteristics during the different time periods

	Group 1 (*N* = 308)	Group 2 (*N* = 183)	Group 3 (*N* = 204)	Group 4 (*N* = 101)	Group 5 (*N* = 232)
Age at randomisation (years) Mean [SD]	11.6 [3.1]	11.9 [3.2]	12.4 [3]	12.1 [3.3]	11.7 [3.1]
Female sex, *n* (%)	163 (53%)	94 (51%)	131 (64%)	61 (60%)	136 (59%)
White ethnicity, *n* (%)	258 (84%)	153 (85%)	169 (84%)	83 (84%)	205 (91%)
Missing (*n*)	2	4	2	2	7
Previous referral to CAMHS, *n* (%)	94 (31%)	63 (35%)	58 (29%)	35 (35%)	75 (33%)
Missing (*n*)	3	5	2	1	6

**Fig. 2:**
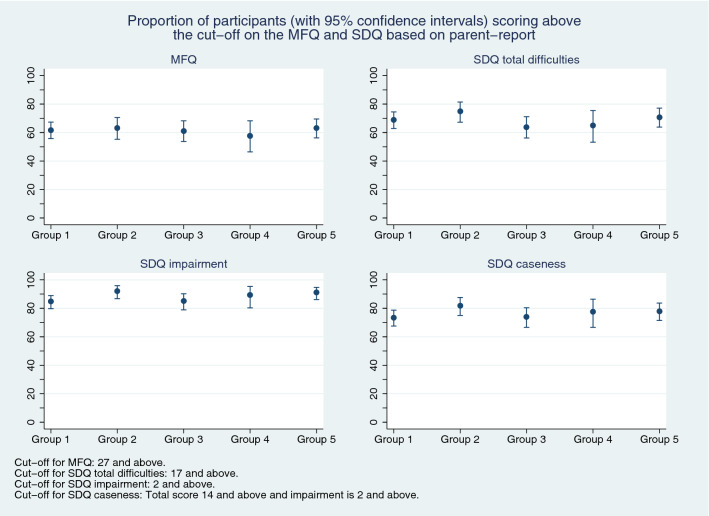
Proportion of participants (with 95% confidence intervals) scoring above the cutoff on the MFQ and SDQ measures, based on parent-report

**Fig. 3:**
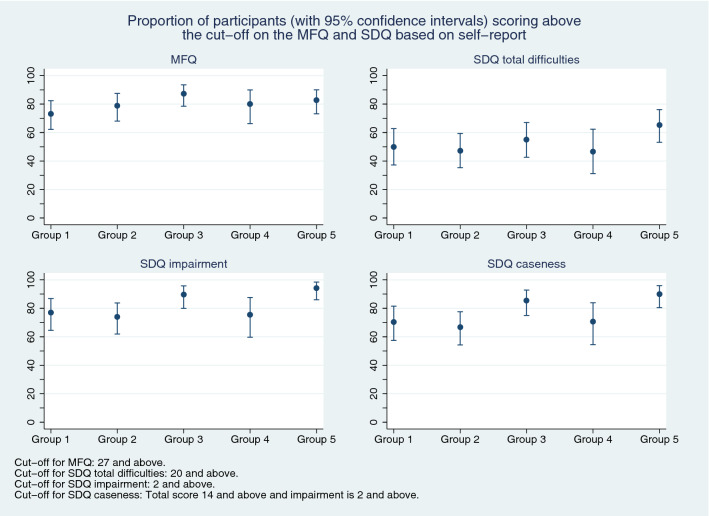
Proportion of participants (with 95% confidence intervals) scoring above the cutoff on the MFQ and SDQ measures, based on self-report

On the MFQ, between 62% (parent-report) and 81% (self-report) of CYP scored above the cut-point for depression. Parent-reported SDQ scores indicated that 82% of CYP scored above the cut-point for emotional problems and almost half had hyperactivity/inattention and conduct problems. Self-report confirmed that emotional problems and hyperactivity/inattention were prominent. Parent- and self- reported scores on the mental health measures are shown in Table 2 and Figs. 2, 3. According to both informants, most CYP had functional impairment and met ‘caseness’ criteria.

**Table 2:** MFQ and SDQ data, by respondent type, during the different time periods

	CYP self-ratings					Parent ratings					
	Group 1 (*N* = 90) Mean [SD] *n* (%)	Group 2 (*N* = 82) Mean [SD] *n* (%)	Group 3 (*N* = 91) Mean [SD] *n* (%)	Group 4 (*N* = 51) Mean [SD] *n* (%)	Group 5 (*N* = 93) Mean [SD] *n* (%)	Group 1 (*N* = 289) Mean [SD] *n* (%)		Group 2 (*N* = 166) Mean [SD] *n* (%)	Group 3 (*N* = 186) Mean [SD] *n* (%)	Group 4 (*N* = 85) Mean [SD] *n* (%)	Group 5 (*N* = 215) Mean [SD] *n* (%)
MFQ	36.9 [14]	35.6 [12.7]	40.1 [11.2]	38.7 [13.5]	40 [13.4]	31.8 [14.3]		31.4 [12.3]	31.1 [14]	31.8 [13.2]	31.9 [13.4]
*n*	82	76	87	50	87	287		163	183	85	214
Prosocial behaviour (SDQ)	6.5 [1.9]	6.5 [2.4]	7.1 [2.4]	7.2 [1.9]	6.6 [2.1]	6.4 [2.8]		6.2 [2.7]	6.4 [2.7]	6.1 [2.5]	6.2 [2.5]
Scoring above cutoff	9 (14%)	16 (22%)	13 (19%)	4 (9%)	11 (15%)	60 (23%)		47 (30%)	44 (26%)	22 (29%)	51 (27%)
*n*	64	72	69	43	72	257		155	169	77	192
Emotional problems (SDQ)	6.8 [2.3]	6.2 [2.3]	7.5 [2.1]	6.9 [2.5]	7.2 [2.1]	6.6 [2.6]		6.6 [2.2]	6.8 [2.5]	6.5 [2.6]	6.8 [2.4]
Scoring above cutoff	37 (58%)	33 (46%)	52 (75%)	29 (67%)	48 (67%)	208 (81%)		127 (82%)	139 (82%)	60 (78%)	159 (83%)
*n*	64	72	69	43	72	257		155	169	77	192
Conduct problems (SDQ)	2.9 [2]	3.1 [2.2]	2.7 [2.1]	2.7 [2.5]	3.3 [2]	3.6 [2.5]		3.8 [2.6]	3.3 [2.4]	3.3 [2.5]	3.5 [2.3]
Scoring above cutoff	13 (20%)	16 (22%)	13 (19%)	10 (23%)	19 (26%)	126 (49%)		74 (48%)	68 (40%)	29 (38%)	88 (46%)
*n*	64	72	69	43	72	257		155	169	77	192
Hyperactivity/inattention (SDQ)	6.1 [2.5]	6.2 [2.5]	6 [2.2]	6.2 [2.7]	6.8 [2.2]	5.9 [2.8]		6.3 [2.6]	5.7 [2.6]	6.1 [2.8]	6.1 [2.8]
Scoring above cutoff	31 (48%)	30 (42%)	29 (42%)	21 (49%)	41 (57%)	114 (44%)		79 (51%)	70 (41%)	35 (45%)	94 (49%)
*n*	64	72	69	43	72	257		155	169	77	192
Peer problems (SDQ)	3.6 [2.1]	3.9 [2.4]	3.7 [2.1]	3.3 [2]	4.1 [1.9]	3.8 [2.3]		4.2 [2.6]	3.6 [2.2]	3.8 [2.3]	3.9 [2.4]
Scoring above cutoff	13 (20%)	17 (24%)	15 (22%)	8 (19%)	19 (26%)	129 (50%)		93 (60%)	86 (51%)	42 (55%)	108 (56%)
*n*	64	72	69	43	72	257		155	169	77	192
Total difficulties (SDQ)	19.5 [5.6]	19.3 [6.5]	19.8 [5.7]	19.1 [5.8]	21.4 [4.9]	19.9 [7.3]		20.8 [6.9]	19.4 [6.6]	19.7 [6.9]	20.3 [6.9]
*n*	64	72	69	43	72	257		155	169	77	192
Impairment (SDQ)	4.1 [2.7]	3.9 [2.6]	4.2 [2.6]	4.2 [2.7]	4.9 [2.4]	5.1 [3]		5.4 [2.8]	5 [2.9]	5.5 [2.7]	5.7 [3]
*n*	61	69	68	41	70	256		154	169	76	191

MFQ collected prior to randomisation; however, there are some missing data due to some secondary participants not providing questionnaire data. SDQ collected immediately post-randomisation so there are some missing data due to some primary and secondary participants not providing questionnaire data (see “Methods”)

*CYP* Children and Young People, *MFQ* Mood and Feelings Questionnaire, *SDQ* Strengths and Difficulties Questionnaire

### Group differences

Figures 2 and 3 show the proportions scoring above cutoff thresholds on the MFQ and SDQ total difficulties, impairment and caseness measures, by informant. In terms of parent-report, although point-estimates differed slightly between the five groups there was no consistent pattern and confidence intervals overlapped (Fig. 2). Overall, there was a broadly similar pattern according to self-report (Fig. 3)—however, compared with Group 1 (the pre-pandemic group), self-reported emotional problems, depression, functional impairment and caseness may have been slightly higher amongst CYP recruited in the two periods after schools had re-opened (Groups 3 and 5). In particular, functional impairment and caseness were greater in Group 5 than Group 2 (i.e. confidence intervals did not overlap).

## Discussion

### Main findings

Unlike studies which have focussed on community-based samples [6–9, 21], this investigation of CYP with emotional difficulties referred for specialist mental health care revealed no strong evidence of differences in the severity or impact of emotional or wider mental health difficulties between those who had been referred before or following the onset of the Covid-19 pandemic. However, amongst those referred to CAMHS after schools had re-opened following periods of closures, young people’s self-reports were suggestive of higher levels of emotional problems, depression, impairment and caseness. This finding might reflect possible greater worries and stresses for young people in relation to their return to school after an up-to-6-month gap and associated pressures around academic learning, re-establishing friendships and peer relationships, and re-adjusting to and managing structured daily routines [3–5].

In particular, there was a possible suggestion that the uncertainties and disruptions associated with a second cycle of school closures and re-openings (affecting those in Group 5) might have been particularly difficult for young people. According to their own reports, they were affected more in terms of functional impairment (reflecting distress and impact on day-to-day functioning) rather than greater severity of emotional symptoms or depression. It is possible that young people may perceive functional impairment differently during periods of school closure, for example referencing their day-to-day functioning against their peer group. Although the study design precludes causal attributions, it could be speculated that perhaps the cumulative effects of repeated lockdowns and associated disruptions to daily life, social isolation, disrupted access to learning and education, and subsequent re-adjustments to in-person schooling may have had a detrimental effect on young people’s ability to manage usual day-to-day expectations and demands. Furthermore, the return to school and possible exposure to experiences that are perceived as stressful or adverse might not have been coupled with the full re-establishment of school-based pastoral and wellbeing support.

Although much research suggests an increase in emotional problems in CYP since the pandemic started [6–9, 21–23], this is a not a universal finding and the situation may be more nuanced [24–27]. Some CYP may have experienced lockdown, school closures and the opportunity to spend more time at home with improved family relationships and support as a respite from their usual daily stresses [28, 29]. For example, improved psychological wellbeing during the first lockdown was found to be associated with less exposure to bullying [29]. A UK community-based study of 10–16-year-olds suggests that levels of pre-existing difficulties may have a possible moderator effect—CYP who had relatively high levels of mental health problems before the pandemic reported improvements in their mental health whereas the converse was found for those with relatively low levels of pre-pandemic mental health problems [30]. A longitudinal study in the Netherlands involving CYP with clinically significant mental health problems (identified through school-based screening before the pandemic) showed a reduction in symptomatology when they were re-assessed in January 2021 [24]. In contrast, in our sample of CYP who had been recently referred to CAMHS, we found no major differences in the severity of mental health difficulties in directly comparable pre-pandemic and in-pandemic groups as a whole, although self-reported impairment might have increased slightly in the periods following school re-openings. However, it is possible that our data were constrained by a ceiling effect, whereby most CYP with emotional difficulties who are clinically referred score high for depression, other mental health difficulties and functional impairment at the point of referral (possibly driving the referral) and any further exacerbations might be difficult to demonstrate through these measures. Service issues for the healthcare system might also have been a factor with an initial reduction in referrals to CAMHS during the early phases of the pandemic and although it is possible that our findings might reflect the possible consequences of delays in referrals being made, this possibility seems unlikely as parent-rated difficulties did not differ across the time points and parental perceptions of difficulties are strong determinants of help-seeking [31]. It might be that any deterioration manifests instead through greater acuity and urgency of clinical presentations, for example through increased suicidal ideation or self-harm [32], leading to urgent or crisis referrals. A study of referral patterns in Ireland highlighted an increase in the number of urgent referrals in the September–November period during 2020 compared with previous years [33].

### Methodological issues

This study has several key strengths. First, the timing of the study: data collection commenced in 2019 and continued through into the pandemic period with participant recruitment maintained throughout—our study spans a 22-month period from 2019 to 2021. Second, we recruited a nationally generalisable sample of clinically referred CYP, across five large sites, who were assessed using the same methodology across pre-pandemic and in-pandemic periods. To our knowledge, no studies have investigated differences in mental health symptoms, impairment and caseness between distinct but comparable samples of recently referred CYP over this time period. Third, we used consistent, reliable and validated mental health measures completed by more than one informant. Fourth, demographic characteristics of participants were similar across the different time phases with no obvious selection biases.

In terms of limitations, this study reflects routine (not urgent/emergency) referrals to CAMHS and does not yield information about changes in the numbers, acuity or complexity of referrals over the time period under investigation. The sample size was modest (although exceeded 1000), reflecting a small proportion of the population of CYP referred to CAMHS during the study period. We were not able to look at differences by presenting problems. Although descriptive analyses might limit the interpretation of the findings, our approach to the analyses can be justified as baseline characteristics of the participants recruited into the STADIA trial were similar across the pre-pandemic and each of the in-pandemic phases, and formal analyses that adjusted for these baseline characteristics were not meaningfully different to unadjusted analyses. As a repeated series of cross-sectional data comparisons over different periods of time, in effect comparing similar samples recruited before and after key cutoff dates, our study does not inform about within-individual change over time. However, although longitudinal research is useful in investigating possible causation, there can also be potential difficulties with interpreting changes over time amongst samples of recently referred cases because of likely regression to the mean with problems tending to be at their most severe at the point of referral.

### Implications

Overall, our findings can provide some reassurance to referrers, clinicians and mental health service providers and commissioners/funders that, amongst routine referrals of CYP in the first 16 months of the pandemic, there was no overall increase in symptom severity at the point of referral. However, there was a suggestion that self-rated functional impairment was slightly elevated amongst those referred in the periods after schools had re-opened, highlighting the importance of asking young people directly and giving particular prominence to their perspectives and lived experiences. Hence, it is important that clinicians, parents and teachers are aware of the impacts of the pandemic-related cycles of school closures and re-openings on the mental health of clinically referred CYP. In terms of research implications, additional research is required to investigate whether urgent and emergency referrals have changed in severity and complexity over this same time. Further research is also needed to assess longer-term impacts as well as the impact of any future unanticipated full or partial school closures or recurrent requests to quarantine or self-isolate at home, which may result in considerable additional disruption to routine and structure. It is vital for CAMHS and schools to have close links to best support CYP’s mental health and wellbeing, particularly when returning to school after periods of closure.

## Data Availability

The data presented here are baseline data from the STADIA randomised controlled trial. Datasets containing individual participant data analysed in this paper will be available upon request from the NCTU (ctu@nottingham.ac.uk) a minimum of 6 months after publication of the main trial results i.e. between-group comparisons of primary and secondary outcomes. Access to the data will be subject to review of a data sharing and use request by a committee including the Chief Investigator and Sponsor and will only be granted upon receipt of an approved protocol and a data sharing and use agreement. Any data shared will be pseudo-anonymized which may impact on the reproducibility of published analyses.

## References

[1] Holmes EA, O’Connor RC, Perry VH, Tracy I, Wessely S, Arseneault L, Ballard C, Christensen H, Cohen-Silver R, Everall I, Ford T, John A, Kabir T, King K, Madan I, Michie S, Przybylski AK, Shafran R, Sweeney A, Worthman CM, Yardley L, Cowan K, Cope C, Hotopf M, Bullmore E (2020). Multidisciplinary research priorities for the COVID-19 pandemic: a call for action for mental health science. Lancet Psychiatry.

[2] Young Minds (2020) Coronavirus: impact on young people with mental health needs https://www.youngminds.org.uk/media/01epl0t1/coronavirus-report-spring-2020.pdf. Accessed 5 July 2022

[3] Winter R, Lavis A (2021). The impact of COVID-19 on Young people's mental health in the UK: key insights from social media using online ethnography. Int J Environ Res Public Health.

[4] McKinlay AR, May T, Dawes J, Fancourt D, Burton A (2022). 'You're just there, alone in your room with your thoughts': a qualitative study about the psychosocial impact of the COVID-19 pandemic among young people living in the UK. BMJ Open.

[5] Coetzee BJ, Gericke H, Human S, Stallard P, Loades M (2022). How young people experienced COVID-19 disease containment measures in the Western Cape, South Africa: a qualitative study including the perspectives of young people, their parents, teachers and school counsellors. Psychol Psychother.

[6] Bignardi G, Dalmaijer ES, Anwyl-Irvine AL, Smith TA, Siugzdaite R, Uh S, Astle DE (2020). Longitudinal increases in childhood depression symptoms during the COVID-19 lockdown. Arch Dis Child.

[7] Wright N, Hill J, Sharp H, Pickles A (2021). Interplay between long-term vulnerability and new risk: Young adolescent and maternal mental health immediately before and during the COVID-19 pandemic. JCPP Adv.

[8] Magson NR, Freeman JYA, Rapee RM, Richardson CE, Oar EL, Fardouly J (2021). Risk and protective factors for prospective changes in adolescent mental health during the COVID-19 pandemic. J Youth Adolesc.

[9] De France K, Hancock GR, Stack DM, Serbin LA, Hollenstein T (2022). The mental health implications of COVID-19 for adolescents: follow-up of a four-wave longitudinal study during the pandemic. Am Psychol.

[10] Vizard T, Sadler K, Ford T, Newlove-Delgado T, McManus S, Marcheselli F, Davis J, Williams T, Leach C, Mandalia D, Cartwright C (2020) Mental health of children and Young people in England, 2020: Wave 1 follow up to the 2017 Survey. https://files.digital.nhs.uk/CB/C41981/mhcyp_2020_rep.pdf. Accessed 5 July 2022

[11] Racine N, McArthur BA, Cooke JE, Eirich R, Zhu J, Madigan S (2021). Global prevalence of depressive and anxiety symptoms in children and adolescents during COVID-19: a meta-analysis. JAMA Pediatr.

[12] Goodman R, Ford T, Richards H, Gatward R, Meltzer H (2000). The development and well-being assessment: description and initial validation of an integrated assessment of child and adolescent psychopathology. J Child Psychol Psychiatry.

[13] Day F, Wyatt L, Bhardwaj A, Dubicka B, Ewart C, Gledhill J, James M, Lang A, Marshall T, Montgomery A, Reynolds S, Sprange K, Thomson L, Bradley E, Lathe J, Newman K, Partlett C, Starr K, Sayal K (2022). STAndardised DIagnostic Assessment for children and young people with emotional difficulties (STADIA): protocol for a multicentre randomised controlled trial. BMJ Open.

[14] Angold A, Costello EJ, Messer SC, Pickles A (1995). Development of a short questionnaire for use in epidemiological studies of depression in children and adolescents. Int J Methods Psychiatr Res.

[15] Wood A, Kroll L, Moore A, Harrington R (1995). Properties of the mood and feelings questionnaire in adolescent psychiatric outpatients: a research note. J Child Psychol Psychiatry.

[16] Daviss WB, Birmaher B, Melham NA, Axelson DA, Michaels SM, Brent DA (2006). Criterion validity of the mood and feelings questionnaire for depressive episodes in clinic and non-clinic subjects. J Child Psychol Psychiatry.

[17] Child Outcomes Research Consortium (CORC) (2002) https://www.corc.uk.net/outcome-experience-measures/. Accessed 5 July 2022

[18] Goodman R (1999). The extended version of the strengths and difficulties questionnaire: as a guide to child psychiatric caseness and consequent burden. J Child Psychol Psychiatry.

[19] Goodman R (2001). Psychometric properties of the strengths and difficulties questionnaire. J Am Acad Child Adolesc Psychiatry.

[20] Sayal K, Taylor E (2004). Detection of child mental health disorders by general practitioners. Br J Gen Pract.

[21] Thorisdottir IE, Asgeirsdottir BB, Kristjansson AL, Valdimarsdottir HB, Jonsdottir-Tolgyes EM, Sigfusson J, Allegrante JR, Sigfusdottir ID, Halldorsdottir T (2021). Depressive symptoms, mental wellbeing, and substance use among adolescents before and during the COVID-19 pandemic in Iceland: a longitudinal, population-based study. Lancet Psychiatry.

[22] Moore G, Anthony R, Angel L, Hawkins J, Morgan K, Copeland L, Murphy S, Van Godwin J, Shendorovich Y (2022). Mental health and life satisfaction among 10–11-year-olds in Wales, before and one year after onset of the COVID-19 pandemic. BMC Public Health.

[23] Luijten MAJ, van Muilekom MM, Teela L, Polderman TJC, Terwee CB, Kijlmans J, Klaufus L, Popma A, Oostrom KJ, van Oers HA, Haverman L (2021). The impact of lockdown during the COVID-19 pandemic on mental and social health of children and adolescents. Qual Life Res.

[24] Bouter DC, Zarchev M, de Neve-Ethoven NGM, Ravensbergen SJ, Kamperman AM, Hoogendijk WJG, Grootendorst-van Mil NH (2022). A longitudinal study of mental health in at-risk adolescents before and during the COVID-19 pandemic. Eur Child Adolesc Psychiatry.

[25] van der Velden PG, van Bakel HJA, Das M (2022). Mental health problems among Dutch adolescents of the general population before and 9 months after the COVID-19 outbreak: a longitudinal cohort study. Psychiatry Res.

[26] Ertanir B, Kassis W, Garrote A (2021). Longitudinal changes in Swiss adolescent's mental health outcomes from before and during the COVID-19 pandemic. Int J Environ Res Public Health.

[27] Belanger RE, Patte KA, Leatherdale ST, Gansaonré RJ, Haddad S (2021). An impact analysis of the early months of the COVID-19 pandemic on mental health in a prospective cohort of Canadian adolescents. J Adolesc Health.

[28] Clemens V, Deschamps P, Fegert JM, Anagnostopoulos D, Bailey S, Doyle M, Eliez S, Hansen AS, Hebebrand J, Hillegers M, Jacobs B, Karwautz A, Kiss E, Kotsis K, Kumperscak HG, Pejovic-Milovancevic M, Christensen AMR, Raynaud JP, Westerinen H, Visnapuu-Bernadt P (2020). Potential effects of “social” distancing measures and school lockdown on child and adolescent mental health. Eur Child Adolesc Psychiatry.

[29] Soneson E, Puntis S, Chapman N, Mansfield KL, Jones PB, Fazel M (2022). Happier during lockdown: a descriptive analysis of self-reported wellbeing in 17,000 UK school students during Covid-19 lockdown. Eur Child Adolesc Psychiatry.

[30] Hu Y, Qian Y (2021). COVID-19 and adolescent mental health in the United Kingdom. J Adolesc Health.

[31] Sayal K (2006). Annotation: pathways to care for children with mental health problems. J Child Psychol Psychiatry.

[32] Mayne SL, Hannan C, Davis M, Young JF, Kelly MK, Powell M, Dalembert G, McPeak KE, Jenssen BP, Fiks AG (2021). COVID-19 and adolescent depression and suicide risk screening outcomes. Pediatrics.

[33] McNicholas F, Kelleher I, Hedderman E, Lynch F, Healy E, Thornton T, Barry E, Kelly L, McDonald J, Holmes K, Kavanagh G, Migone M (2021). Referral patterns for specialist child and adolescent mental health services in the Republic of Ireland during the COVID-19 pandemic compared with 2019 and 2018. BJPsych Open.

